# A reply to “Fundamental issues in epistemic injustice in healthcare” by Nielsen, Nordgaard & Henriksen (2025): Corresponding author:

**DOI:** 10.1007/s11019-026-10392-w

**Published:** 2026-08-18

**Authors:** Eleanor A. Byrne, Havi Carel, Fred Cooper, Dan Degerman, Alice Monypenny, Matthew Broome

**Affiliations:** 1 School of Humanities, University of Nottingham, University Park, NG7 2RD Nottingham, United Kingdom; 2 University of Bristol, Bristol, United Kingdom; 3 University of Nottingham, Nottingham, United Kingdom; 4 University of Birmingham, Birmingham, United Kingdom

**Keywords:** Epistemic injustice, Healthcare, Illness, Testimonial injustice, Hermeneutic injustice

## Abstract

Nielsen et al. (2025) have recently argued that the study of epistemic injustice in healthcare is subject to issues that, left unaddressed, threaten the ongoing research programme. One of their central worries is a lack of appropriate empirical evidence to support claims about the ubiquity and seriousness of these epistemic injustices which are commonly endorsed and rehearsed in publications on the topic. As researchers on **the project Epistemic Injustice in Healthcare (EPIC), ** we offer responses to their concerns. While we sympathise with and develop some of their concerns, we express caution regarding others. We argue for an ongoing research agenda which is empirically-informed but maintains a pluralistic approach to research methods and approaches. This, we argue, is necessary in order to accommodate the complex social phenomenon of epistemic injustice in healthcare contexts.

## An overview of NNH’s critique

Research into epistemic injustice in healthcare has gained significant traction over the past decade. Nielsen et al. (2025) (hereafter, NNH) are concerned that this now highly active research area is subject to problems which are sufficiently serious to require pause and reconsideration. They classify these problems as ‘scientific’, ‘conceptual’, or ‘theoretical’.

What they call their ‘scientific’ and ‘conceptual’ critiques are tightly bound. They involve the worries that: (a) there is insufficient empirical data to substantiate widely endorsed claims about the ubiquity of epistemic injustice in healthcare, and (b) these empirically unsubstantiated claims have come to function as unquestioned assumptions of this research programme. Furthermore, if these empirical claims turn out, on examination, to be false, then the need to study epistemic injustice in healthcare is called into question. NNH worry that repeated assertions of the prevalence of epistemic injustice without strong qualitative and quantitative evidence is a problem so big that it calls into question the clinical relevance of the entire research programme. Framed as a further conceptual critique, NNH also worry that much of the contemporary research works with an unnecessarily “antagonistic schema” pitting patients against clinicians – in effect, constructing a “straw clinician” (pp. 293–298).

NNH’s theoretical critique concerns alleged ambiguities about how epistemic injustice is measured and defined. Is epistemic injustice defined by (i) the experiences of the patient, (ii) the beliefs and actions of healthcare practitioners, (iii) some combination of these, or (iv) something else? NNH adopt Miranda Fricker’s original claim that prejudice and negative stereotyping are integral to testimonial injustice (indeed this is the only form of epistemic injustice that NNH address). In a later section of their paper, NNH note that contrary to Fricker’s framework, subsequent work on epistemic injustice has instead adopted the experience of the subject as theoretically central. NNH consider this a troubling inconsistency that puts pressure on our most basic understanding of what epistemic injustice is. This then raises the question: to assert that an epistemic injustice has taken place, do we need to be able to make and defend claims about the inner motivational states of healthcare practitioners? Or is it sufficient to simply argue that epistemic injustice has taken place simply because the patient experiences it as such? For NNH, this ambiguity demands resolution if work on epistemic injustices in healthcare is to achieve a desired level of theoretical clarity.

NNH contend that failure to resolve these theoretical ambiguities and evidential concerns would warrant scrutiny of the entire epistemic injustice in healthcare research programme. This is no small thing: the influence of the epistemic injustice in healthcare research programme has been wide-ranging, from contesting dominant views on diagnostic categories (Bueter 2019; Wardrope 1987), to critiquing processes and behaviours within healthcare institutions (Bueter and Jukola 2025; Michaels 2021; Carel and Kidd 2021), and questioning metaphysical views such as naturalism that underpin medical thought, training and education (Kidd and Carel 2018; Hall 2017). More concretely, the concept of epistemic injustice has made its way into training resources, been given uptake by patient organisations, advocacy groups, health regulators and so on (e.g. **EPIC 2025**; Care Quality Commission 2023; Degerman et al. 2025a, b). If the foundations of the concept fare badly under scrutiny, it raises the question whether this research programme is effective in its aim to mitigate at least some of the suffering of patients associated with their epistemic treatment.

While in practice the scientific, conceptual and theoretical are intimately related, we organise our discussion around the distinctions NNH set out: section two responds to the scientific and conceptual, and section three to the theoretical. We address the issues throughout the two sections, with minimal but unavoidable repetition and cross referencing to address this. Each section first states NNH’s critique, followed by our responses.

## Scientific and conceptual problems

NNH state that “the field is confronted by a compelling lack of empirical evidence” (p. 292). They emphasise that research into epistemic injustice in healthcare ought to be interdisciplinary, sensitive to empirical evidence, and grounded in clinical reality. Without this, the research risks becoming “sterile” and lacking clinical relevance or impact (p. 293). More specifically, NNH argue that the field urgently needs qualitative and quantitative empirical evidence to support the claims that epistemic injustice in healthcare is as ubiquitous as the literature would suggest. They claim, however, that before empirical studies can be conducted, we must (i) establish a method for measuring epistemic injustice and (ii) validate that measure and ensure that it measures the construct of epistemic injustice and nothing else (ibid.). NNH consider it problematic that the field appears to over-rely on a limited set of particular “case studies” (p. 292), vignettes or anecdotes, and they argue that the field should develop qualitative and quantitative techniques to systematically document the prevalence and severity of epistemic injustice in healthcare. Moreover, according to NNH, the claims made in the epistemic injustice literature require empirical testing via large-scale empirical studies using quantitative methodology; without this, they claim, the field is subject to major scientific limitations. In what follows, we focus on specific claims and suggestions made by NNH and argue that while some of their critique is well-placed, other aspects of it require caution.

### The role of empirical data

NNH argue that until sophisticated qualitative and quantitative measures for investigating the prevalence and severity of epistemic injustice in healthcare have been established, theoretical work in the field should not continue. They assert that the field relies “only on case studies and theoretical conjectures” and that such evidence does not support claims about how widespread or serious the problem of epistemic injustice is in healthcare. According to NNH, that would require “large-scale empirical studies using quantitative methodology” (p. 292). We agree that robust measurement techniques which have been demonstrated to adequately capture epistemic injustice can have a part to play in a pluralistic approach to how the phenomenon can be evidenced. However, we express caution about (i) endorsing a hierarchical conception of evidence and (ii) the claim that the value of the field is contingent upon large-scale quantitative research. We reject the methodological generalisation implicit in evaluating research on epistemic injustice according to evidential standards developed for a different class of clinical research questions. The tendency to treat methodologies associated with biomedical intervention research as the ‘gold standard’ for all forms of clinical and healthcare research has long been criticised by clinical epidemiologists and philosophers of science (Feinstein and Horwitz 1997; Askheim et al. 2017). More recently, related concerns have been articulated in critiques of ‘methodological fetishism’, referring to the privileging of particular methodological approaches with insufficient sensitivity to their scope and limitations (Greenhalgh 2012; Færge et al. 2026).

More specifically, NNH are concerned about the field’s reliance on “case studies” given that interpretations of case studies are highly vulnerable to researcher bias (p. 292). The worry here is that those who are researching epistemic injustice in healthcare contexts are primed to see instantiations of it where an interpersonal exchange may be plausibly interpreted differently. In response, we suggest that the risk of bias (which is always present in qualitative research and more generally when we interpret the words of others) should not be taken to undermine the value and power of the contribution of case studies, anecdotes, patient reports, formal complaints, and testimonies, as forms of evidence (Forrester 2016). The question of bias, significant though it is, should not paralyse research or undermine ways of gaining knowledge. Instead the problem of bias should – and is – recognised, studied and reflected on by researchers as part of their research process.

Additionally, researchers in the humanities and social sciences use examples and case studies, as well as a host of other methods (thought experiments, archival work, hypothetical scenarios) to build and test empirically-informed arguments and intuitions (Greenhalgh 2016; Williamson 2007). Moreover, it is not clear how the question of bias concerns case studies considerably more than it does other forms of empirical investigation which is, after all, always situated and shaped by the nature of the investigation – its investigators, tools, concepts and so on (Hacking 1983; Longino 1990; Chang 2012). The idea of the objective researcher, in the natural and clinical sciences as well as in the humanities, is a concept that has long been under scrutiny, generating useful reflections on the stance of the researcher (Millard 2020; see also Michaels 2021).

NNH are right that there are currently few systematic empirical studies of the sources, forms, prevalence, and effects of epistemic injustices in healthcare contexts. However, some work is emerging (Jongsma et al. 2017; Harris et al. 2022). Current work building questions about epistemic injustice into broader questionnaires and patient surveys is also underway in several countries. Indeed, this need for empirical investigation was one impetus for **project EPIC**.^1^ We now turn to examine how empirical research tools could be developed and call for a broader conception of evidence than NNH endorse.

The central question, we suggest, is: what kinds of evidence are relevant in helping to track and ameliorate epistemic injustice? There is a wealth of methods and resources available to us, and this is not a shortcoming: epistemic injustice is an inherently social phenomenon, and because such phenomena are multifaceted and complex, a broad array of methods is needed to bring to light different relevant features of such phenomena (e.g. epistemic, linguistic, affective, political, etc.). The breadth of available methodologies invites reflection about the criteria for evidential adequacy, relevance, and priority. Moreover, different methodologies can shed light on different aspects of epistemic injustice; for instance, archival research can be indispensable in illuminating historical and cultural context, whereas empirical qualitative work in clinical settings which can shed light on contemporary clinical contexts. As this discussion shows, it is important to resist aligning oneself with a hierarchical or overly scientistic conception of evidence that would deprioritise evidence from a broad range of disciplines within humanities and social science (Raphael 2013; Murad et al. 2016).

Moreover, many illnesses cannot be well (if at all) captured or measured solely within the confines of our current best quantitative empirical techniques: many conditions, such as psychiatric and functional disorders, have no consistently identifiable biomarkers. We should be wary of reasoning which would imply that such conditions are not ‘real’, since they cannot be ‘verified’ or ‘proven’ with quantitative empirical methods—a misstep that has been critiqued for decades (Wessely 1997; Greenhalgh et al. 2018; Byrne 2022; Hunt et al. 2023). Moreover, empirical historical research into the life cycles of social, medical, and public health issues demonstrates how measurement scales and ratings exert influence over the credibility of other kinds of evidence (McGuire 2016).

Quantitative research is certainly imperative in many research areas; it can be invaluable in enumerating phenomena identified and tracked through lived experience, case reports, arts and humanities research, or observations from practice. It can also, for example, help systematically track and draw connections between phenomena. However, we ought to resist aligning ourselves with the view that quantitative evidence is a crucial preliminary step necessary in order to conduct research into epistemic injustice at all. The evidence we have about epistemic injustice in healthcare is robust enough to drive empirical hypotheses. Therefore, we see no reason to withhold research until these hypotheses are supported by quantitative evidence of the scale NNH suggest is required; there should be an ongoing iterative exchange between the empirical and the conceptual, so concepts are stress-tested by being put into specific contexts and examined and refined in light of real world situations and examples.

Additionally, we have relevant evidence that has been collected by other means, for example, by sets of questions asked by the Care Quality Commission (CQC 2025), including questions like ‘When doctors/nurses spoke about your care in front of you, were you included in the conversation?’ and ‘To what extent did staff looking after you involve you in decisions about your care and treatment?’. Further evidence is currently collected via a large Norwegian online questionnaire, showing a strong correlation between patient reported epistemic injustice (measured through **a project EPIC questionnaire)** and other measures, such as existential isolation, loneliness, and depression, as well as a strong negative correlation with social support.^2^

Counter to NNH’s approach, we claim that epistemic injustice is not a highly abstract theoretical construct, the validation of which depends on a complex theory. It is an intuitive, readily understood phenomenon, expressible in everyday language, even if, importantly, its philosophical articulation raises important questions about its underpinning structure and causes. One reason for the wide uptake of epistemic injustice literature in health research is that it offers a set of robust interpretive resources to understand and systematise problems that researchers were already substantially engaged with but lacked a clear name for. Indeed, the remarkable uptake of the concept across many disciplines, provides robust evidence of the intuitive appeal of the concept (for a full bibliometric analysis see Báez-Vizcaíno 2024; see also Kidd et al. 2019: Part V; Carel and Kidd 2021, 2024).

Despite the term only coming into existence in the early 2000s, there are now hundreds of published academic and non-academic outputs documenting it, refining the concept and applying it in areas as diverse as environmental science and education. In terms of disciplines, almost half the publications are in health research or in social science, the two areas in which epistemic injustice in healthcare would be primarily studied (Báez-Vizcaíno 2024). Researchers have used testimonies, memoirs, interviews, and other qualitative data to identify and dissect epistemic injustices in a variety of healthcare contexts, including autism (Catala et al. 2021), bipolar disorder (Degerman 2025), borderline personality disorder (Kyratsous and Sanati 2016), gynaecology and obstetrics (FitzGerald 2020; Shabot 2021), chronic fatigue syndrome (Blease et al. 2017; Byrne 2020); Long Covid (Byrne 2022), chronic pain (Buchman et al. 2017), dementia (Spencer 2023), depression (Jackson 2017), disability (Cupples 2020), intersex health (Merrick 2019), voice-hearing (Harris et al. 2022), young people’s mental health (Greenblatt et al. 2024), informal/family caring and female caregivers (Isham et al. 2020; Cooper 2026) and transgender healthcare (Freeman and Stewart 2021). This significant body of work amounts to a collective mapping of systemic problems from different perspectives and requiring different bodies of disciplinary expertise.

Furthermore, systemic changes such as Martha’s rule demonstrate the need for a clear process to amplify epistemic input from carers and families. The rule was developed in response to the death of Martha Mills, who fell off her bike and later died of sepsis in 2021, age 13. Her death, deemed to have been avoidable by a coroner in 2023, triggered a patient safety initiative, giving “patients, families and carers the right to request a rapid review if they are worried that a patient’s condition is getting worse and their concerns are not being responded to.”^3^ This rule recognises the epistemic contributions made by others (e.g. family members) that busy healthcare professionals might otherwise miss. This is also true in the context of psychiatry (Broome 2024).

We suggest that research in the field ought to be interdisciplinary, sensitive to empirical evidence, and grounded in clinical reality. It is also worth thinking strategically about what corners of epistemic injustice in healthcare research remain philosophically interesting to pursue. We agree with Robin McKenna that there is a ‘saturation point’ beyond which articles repeat familiar claims about epistemic injustice applied to a particular medical condition.^4^ We ought to, however, distinguish repetition from degradation. There are still under-developed areas of the research programme which we ought to turn our attention to: perhaps it would be more productive to focus on particular areas within medicine, such as geriatrics or paediatrics, institutional epistemic injustice, comparative research of epistemic injustice in primary, secondary and tertiary care, or how epistemic injustices are distributed across the healthcare sector and globally. Ongoing research in **project EPIC** and beyond has been uncovering unique ways in which epistemic injustice operates, for example, how it might be perpetuated towards nonverbal people, critically ill patients (Carel 2025a, b) or those who suffer from memory loss (e.g. Calabrese, 2025), and what intersectional analyses might reveal about how epistemic injustice affects particular patient groups (e.g. Wiggleton-Little 2026; Byrne and de Boer 2025). Such research can use broader frameworks such as ‘the predicament of patients’ (Kidd and Carel 2021) to study specific elements of pathocentric epistemic injustice (Kidd and Carel 2018a). We suggest that the above offer fresh avenues that merit further study within the epistemic injustice in healthcare programme.

Empirical work – both quantitative and qualitative – can and does help us develop and refine the theoretical concept of epistemic injustice. This dynamic interaction between the theoretical and the empirical can help create further distinctions, challenge others, and develop new concepts which may have greater explanatory power or clinical care utility. The project EPIC programme is based on this iterative process which triangulates philosophical concepts against real-world features, including empirical data, clinical practices, institutional pressures, and other factors needed to develop a full picture of epistemic injustice.^5^ Putting pressure on theoretical concepts in this way, and from multiple disciplinary perspectives using a variety of research methods, should provide robust evidence of the reality of the concept, as well as how it can be operationalised in different contexts.

There are examples of this already: the project EPIC case studies use qualitative interviews to track and document instances of epistemic injustice as reported by psychiatric patients.^6^ Byrne (2020) analysed the concepts of epistemic injustice in relation to CFS/ME and suggested that conceptual impoverishment should trigger a revision of which supposed testimonial injustices are better explained as hermeneutical problems; de Boer (2021) carried out discourse analysis on a CFS/ME patient participation programme in the Netherlands to evidence and theoretically develop this. This shows that empirical work can (and does, slowly and iteratively) engage in dialogue with conceptual work on epistemic injustice.

More broadly, further value is found in interdisciplinary work connecting philosophy as a theoretical discipline to the empirical, clinical, and social sciences. Developing standardised healthcare tools based on philosophical theory has been successful in other areas. For instance, structured interview tools for investigating schizophrenia spectrum disorders – such as the Examination of Anomalous Self Experience (EASE) – have used phenomenological theory to highlight the specificity of self-disorders in schizophrenia in a systematic way. The EASE scale has been shown to have strong internal consistency and inter-rater reliability (Møller et al. 2011), which supports its promise of clinical utility (Burgin et al. 2022). A Covid-19 questionnaire developed by philosophers and phenomenologists has illuminated particular aspects of these experiences informed by phenomenological concepts, such as bodily doubt, trust, and loss of world (Froese et al. 2021; James et al. 2022; Rodrigues et al. 2023). Finally, a scoping review conducted by a multidisciplinary team that includes philosophers shows the extent to which the lack of attunement to, and inclusion of, dementia patients in research permeates the research design itself, building on philosophical work on lived experience (Calabrese et al. 2026).

To conclude this section, consider a concrete example of supposed identity prejudice against patients. NNH argue that to prove the occurrence of identity prejudice amongst clinicians, sophisticated empirical measures need to be developed. Their point is bolstered by pointing towards extant empirical measures into implicit bias, such as the Implicit Association Test (IAT). They suggest that research into epistemic injustice in healthcare could follow suit and take inspiration from such measures. This highlights the point we wish to emphasise. After twenty years of development and refinement, such measures remain the subject of great controversy and critique: prominent researchers still consider IATs to be “noisy measures that add nothing to the information we already get from cheaper self-reports” (Schimmack 2021). The IAT has been criticised for low internal consistency, validity, test-retest reliability, and weak correlation with real-world behaviours (Jost 2018; Meissner et al. 2021; Röhner and Iliescu 2023). Importantly, such limitations do not entail that claims of implicit bias are thereby undermined, only that our measures and evidence require ongoing refinement (Greenwald et al. 2022). We suggest that epistemic injustice should be afforded a similar level of resilience: the limitations of particular measurement tools should prompt refinement, not scepticism about the concept itself.

### The straw clinician

NNH point out that research into epistemic injustice in healthcare operates with a picture of a “straw clinician”: “Rather than picking out features of reality, the claims about clinicians harboring negative stereotypical prejudices toward patients seem to create […] a distorted image of the clinician” (p. 293). Relatedly, NNH raise concerns about the unhelpful use of antagonistic schemas and ‘conceptual pairs’ like ‘victims’ and ‘perpetrators’ (p. 298). We concur that such antagonistic schemas are unhelpful, as some researchers have pointed out (Byrne 2020; Kidd & Carel 2017; Carel and Macnaughton 2012). Moreover, **project EPIC research** has emphasised the plurality of professions, specialisations, and approaches within healthcare, the internal epistemic hierarchies of healthcare institutions, and the varied contexts of healthcare encounters, in order to oppose the reductive appellation ‘the doctor,’ which we see as unhelpful, divorced from the realities of healthcare practice, and promoting an adversarial attitude. The field must steer clear of conceptual pairings which oversimplify the relationships between patients and healthcare professionals.

Healthcare professionals, of course, can also be trapped within problematic or even dysfunctional epistemic systems. For instance, there can be a hierarchy of knowledge and practice where the views of certain clinical disciplines, professional groups, or methodologies are discounted. Recent literature into ‘moral injury’ in psychiatric practice speaks to situations of under-resourcing and burn-out where clinicians feel they are forced to make clinical decisions that conflict with their convictions and ideals about good practice (Broome et al. 2024). Moreover, of course, clinicians can also be patients or advocates for family members with health issues, and as such can have their own views and opinions discounted, when no longer protected by the ‘magic white coat’, as Klitzman (2008) calls it. Sometimes their professional role can cause additional epistemic disadvantages when a patient role is also assumed; for instance, where there is the perception that mental illness is synonymous with incompetency (Ng et al. 2024).

While epistemic injustice can disadvantage both patients and clinicians within the context of an institutional structure, patients are distinctively vulnerable (Carel and Kidd 2021, 2024). They are the ones who are unwell and physically and mentally vulnerable, anxious, and in a state of ill health and suffering. It is patients who need to decide on complex treatment options, which will critically impact on their life. There is a profound difference, therefore, between professional medical engagement and personal engagement, creating an experiential and epistemic rift (Toombs 1987; Carel 2018). This difference should be attended to also within the context of epistemic injustice. Attention to language used can be instructive on this point. Cox and Fritz (2022) point out that much medical language belittles patients, casts them as childlike or passive (“I took their history”, “patient was sent home”), and blames patients (“failed to respond to treatment”). Such language “runs counter to the collaborative relationships [doctors] are trying to foster” (ibid.). It hampers epistemic and communicative attempts by patients, inculcates a sense of guilt and failure, and constitutes patients as epistemically lacking and as failing to be ‘good patients.’

Additionally, health professionals, like all humans, display a high degree of variability in their communication skills, ability to listen, and compassion. This can vary across the professionals and depend on particular interactions with individual patients (Dhairyawan 2024). This variability is and ought to be subject to a benchmark for ‘good enough’ practice and to ensure that skills such as good listening and respect are intrinsic to the professional role, rather than being seen as requiring a trade-off with scientific knowledge and technical skill. The institutional need to safeguard vulnerable patients, and to ensure interactions meet a minimal level of epistemic justice, respect, patient dignity and good quality of discussion and decision making, must underpin this. Therefore, it is insufficient to look solely to the individual ‘straw clinician’ here, since their work must be understood in the context of an institutional culture and processes for ensuring good quality care.

Given the time it has taken for the empirical research to catch up with the theorising, much of the focus within the field has been on the application of the epistemic injustice framework to already recognised failures of communication within healthcare—in many cases drawn from extensive literatures of patient testimonies, memoirs, complaints and media coverage, as well as inquiries and reports into institutional healthcare failings (for discussion of these see Carel and Kidd 2021). This initial focus on the most egregious and easily identified cases in the early waves of the literature, perhaps creates the illusion that the field as a whole has erected a ‘straw clinician’. However, this is only a partial picture, with later work identifying how theories taught within medical education and training, for example naturalism (Goldberg 2017; Kidd and Carel 2018b), contribute to epistemic injustices—thus shifting the focus from individual clinicians to structures such as medical education. Carel and Kidd’s (2021, 2024) recent work on vulnerabilisation and institutional failings analyses the role of institutions, rather than individuals, in causing or exacerbating epistemic injustice, and any analysis of ‘the clinician’ must be understood within their milieu, context, training, and professional culture (cf. Dhairyawan 2024).

The relationship between patient and healthcare professional does perhaps differ from some other examples of epistemic injustice because of the distinct epistemic roles occupied by each party. NNH assert that the ‘doctor-patient relationship is inherently asymmetrical’ due to the one-way care relationship (p. 298). However, it is a mistake to take this asymmetry to indicate a necessary passivity on the part of the patient, or that their epistemic role is confined only to that of an informant regarding symptoms and a recipient of expert testimony (Kidd and Carel 2017). This is especially true in regard to currently under-researched or poorly understood conditions where patients and patient communities occupy an active role in the initiation and generation of new research and direct the course of diagnostic inquiry along with receptive clinicians (Canson 2025).

## Theoretical issues

NNH identify a number of theoretical issues with the application of Fricker’s original framework to epistemic injustice in healthcare research. NNH note that a lot of theoretical work has been done since Fricker’s original analysis, but they limit their critique to this original analysis on the grounds that (i) Fricker’s framework is the core theoretical framework in the field and (ii) Fricker’s original framework can help researchers navigate some of the theoretical issues they identify. Researchers in epistemic injustice clearly owe a vast debt to Fricker’s (2007) pioneering work, but the field of epistemic injustice in healthcare research has developed significantly since, in ways that build on and expand the Frickerian framework (Kidd & Carel 2017). There are, we suggest, details in subsequent work on epistemic injustice in healthcare that helps us address some of the issues that NNH raise.

NNH argue that Fricker is interested in systematic rather than incidental testimonial injustice which involves credibility deficit arising from a “tracker” identity prejudice in the hearer. On Fricker’s original analysis, this is an important feature of epistemic injustice which allows us to demarcate it from cases of epistemic bad luck (Fricker 2007, p. 152). Such instances of epistemic bad luck, perhaps with only incidental identity prejudice or no prejudice at all, do not amount to epistemic injustice even if they are bad or harmful. NNH argue that many cases of epistemic injustice discussed in the literature are not adequately evidenced as satisfying these demarcation criteria. As such, we simply cannot tell if they are cases of epistemic injustice or not. NNH consider various examples from the extant literature to make this point, including a case by Crichton et al. (2017) whereby a young male who was admitted to a psychiatric ward with suspected delusions which later turned out to be true. NNH argue that we are not equipped to state that cases like this involve systematic prejudice; they may just be ‘innocent errors’ or epistemically, but not ethically, culpable instances of poor care.

### Unintentional prejudice and health stigma

NNH write that for a doctor’s deflation of a patient’s credibility to count as testimonial injustice, the deflation must be driven by an unintentional identity prejudice on the part of the doctor: “Testimonial injustice concerns the hearers’ psychology and their biases.” (p. 297). NNH argue that in the types of cases that feature in the literature, the dismissal of patient testimony is often intentional. Moreover, when a dismissal occurs it is unclear whether an identity prejudice is involved and, if so, what the relevant prejudice is. They write:

To make it a case of testimonial injustice, we would again have to assume that we know what drives the clinicians in disregarding parts of patient testimonies, namely, an *unintentional* identity prejudice entertained by the clinician toward the patient instead of an *intentional* relevancy assessment occasioned by medical expertise. By making this assumption we are, of course, skating on thin ice. (p. 297, their emphasis).

They rightly observe that when a doctor dismisses a patient’s testimony, they often do so intentionally in order to filter out clinically irrelevant information. As a doctor observes: ‘patients say a lot of irrelevant things like “when I eat lettuce my elbow hurts.” I have to listen carefully for the important stuff and ignore the rest’ (Carel and Kidd 2014, p. 530). Such intentional relevancy assessments are often necessary and justified, according to NNH (p. 296). First, let us consider NNH’s contention that since such assessments are intentional, they cannot count as an epistemic injustice, which must, again, be driven by an unintentional identity prejudice.

Their argument confuses the presence of a conscious intention with the absence of an unintentional prejudice. There is strong evidence that our explicit and well-reasoned explanations for our actions are sometimes simply post-hoc rationalization of behaviour that clearly had a different, unconscious cause (e.g. Summers 2017). It is, thus, perfectly plausible that a doctor would tell themselves and others that they dismissed what someone is saying because they deemed it clinically irrelevant, even if their actions were actually driven by an unintentional identity prejudice. Such prejudices could be present in at least two ways: discreetly or built into medical concepts and procedures.

To see this, we can consider one of the examples that NNH draw on throughout their paper. It is a real-life case, described by Carel and Kidd, in which a woman who has just given birth is being given vaginal stitches. During the procedure, she cries out in pain, without response. She then cries out again and asks for anaesthesia. This time the doctor replies that they are nearly done and continues. While Carel and Kidd diagnose this as an instance of testimonial injustice, NNH suggest that the more plausible explanation is that the doctor intentionally dismissed the pleas of the patient because they judged it to be in their best interest and, therefore, is not committing an epistemic injustice. That is possible. However, an explicit judgement such as this may equally conceal a discreet identity prejudice operating below the level of explicit awareness – i.e. unintentionally – that leads to the dismissal. For example, the doctor might have an unconscious belief that postpartum women always exaggerate the pain they experience, which leads them to ignore the woman’s cries of pain and pleas for anaesthetic. Another possibility is that the prejudice is present not as a discrete belief that the doctor holds about postpartum women but an assumption built into the medical concepts that they use. It could be, for example, that established procedures for medical stitching involve ignoring patients’ pain because postpartum women have been thought to exaggerate and that this is what leads the doctor to dismiss the woman’s pleas. It is not clear whether this would straightforwardly count as a testimonial injustice on Fricker’s original account. However, the presence of a conscious non-prejudiced intention to dismiss or deflate testimony does not entail that there may not also be an unintentional identity prejudice at play.

NNH also question the basis for claiming that identity prejudices distort doctors’ judgments, particularly in the cases used in the epistemic injustice literature. In fact, they assert that no empirical evidence supports the prevalence of such prejudices. They are right that there are sometimes other explanations for why clinicians treat patient testimonies as they do in the cases discussed in the epistemic injustice literature. However, there is a large body of empirical evidence from health stigma research showing that many doctors hold identity prejudices about people with certain illnesses (Jauch et al. 2023; Ren et al. 2025). NNH gesture towards this evidence, but only to claim that ‘research on epistemic injustice cannot piggyback on empirical stigma research’ if the distinctiveness of the concept is to be preserved (p. 289). We claim that it is not clear why that should be so, provided it is done carefully.

The two phenomena—epistemic injustice and health stigma—are importantly related. Some scales used to explore health stigma among health professionals and other groups explore issues that resonate with the epistemic injustice framework. For instance, the ‘Mental Illness: Clinicians Attitudes Scale’ asks respondents to rate their level of agreement with the following statement: ‘If a person with a mental illness complained of physical symptoms (such as chest pain), I would attribute it to their mental illness’ (Gabbidon et al. 2013). High levels of agreement with this statement within a population of health professionals could be evidence of, at least, a risk of testimonial injustice (for a poignant example of a serious health risk to a psychiatric patient as a result of such beliefs see Saks 2007). However, the fact that health stigma measures explore such questions does not undermine the distinctiveness of epistemic injustice. There is, thus, no reason why research on epistemic injustice in healthcare could not draw on stigma research; indeed, it should and it has.

Nevertheless, we grant that there is a need to conduct more work on epistemic injustice from the healthcare professional’s perspective. Much of the extant research has concentrated on the patient perspective due to its historical and continuing marginalization. This needs to be complemented with research on the factors that may lead health professionals to downgrade or dismiss the contributions of patients.

### The complexity of relevancy assessments

As highlighted in the previous section, relevancy assessments can be necessary, justified, and occasioned by medical expertise. We do not contest the notion that medical expertise can confer particular *types* of epistemic authority, nor do we wish to claim that relevancy assessments are inherently unjust. However, because NNH’s analysis intentionally does not address research into hermeneutic and structural injustice, NNH do not consider more complex cases where these types of epistemic injustices can operate. As such, we suggest that relevancy assessments are often more complex than NNH acknowledge.

Sometimes, healthcare professionals can intentionally make relevancy assessments based on medical expertise, yet medical expertise is limited at least in part *because of* epistemic injustice. The case of the patient who says “when I eat lettuce my elbow hurts” is, arguably, intended as a caricature-like example where the medical irrelevance of the statement should be obvious. As such, it is supposed to be clear that dismissing patient testimony of this kind cannot amount to epistemic injustice.

Ideally, doctors’ relevancy assessments should be sensitive to new and emerging medical knowledge, and as such, those assessments may change over time in response to progress in medical research. However, sometimes the state-of-the-art knowledge about certain conditions is constrained by tracker prejudices (i.e. hermeneutical injustices), not incidental gaps in medical knowledge. For example, medical knowledge (and the uptake of that knowledge) about female health conditions such as endometriosis is, despite increasing rapidly, still poor. A number of researchers have pointed out the role of sexism and misogyny in this conceptual impoverishment (Byrne and de Boer 2025; Cleghorn 2021; **EPIC 2025).**

Besides female health conditions like endometriosis, there are various other chronic and complex health conditions that disproportionately affect women, such as fibromyalgia, Ehlers-Danlos Syndrome (EDS), Post Orthostatic Tachycardia Syndrome (POTS) and Mast Cell Activation Syndrome (MCAS). These conditions occupy a complex position epistemically: they are not strictly ‘unexplained’, yet they are under-explained, with many different conceptions of their pathogenesis and aetiology. The symptom profiles are broad, varied and, to many sufferers and clinicians alike, bewildering at first.

A specific example should motivate our point well. Systemic Nickel Allergy Syndrome (SNAS)—which disproportionately affects women and frequently co-occurs with other conditions like MCAS and EDS—is triggered through contact with or ingestion of nickel, which is found in high concentrations in leafy vegetables. Hence, eating green leafy vegetables like lettuce can trigger symptoms including joint pain (Perez et al. 2015). The relevant point here is how *prima facie* irrelevant patient testimony can in fact be (i) relevant and more importantly (ii) dismissed as irrelevant *because of* hermeneutical gaps that arise because of tracker prejudices like sex and gender. If only (i) obtained, one could argue that cases like these are more like the ‘freak cases’ in which something that seems like a florid delusion, such as seen in schizophrenia, turns out to be true, against all odds. In such cases, according to NNH, we are dealing with non-culpable error rather than epistemic injustice (p. 276). Yet (ii) also obtains. In working with a limited conception of epistemic injustice (i.e. only cases where a clinician unintentionally enacts certain psychological biases) it is not surprising that NNH do not see the applicability of the concept in cases like these. However, hermeneutic injustice, and later the relationship between testimonial and hermeneutic injustice, has been a central feature in epistemic injustice research for many years (Medina 2012, 2017; Catala 2025).

Similarly, structural epistemic injustice can occur prior to the clinician’s intentional relevancy assessment. How patients present information, whether intentionally or not, may be influenced by resistance or dismissal experienced in prior engagements with healthcare professionals. Although NNH note that ‘patients should not be blamed for providing this [irrelevant] information’ (p. 297), patients can become vigilant against judgements regarding their trustworthiness and character more generally. For example, experiences of not being taken seriously by healthcare professionals is not uncommon amongst patients seeking treatment for chronic pain. As a result, some take great care in presenting themselves so as to appear credible (Buchman et al. 2017, p. 36−37), or otherwise withhold certain information (see for example, Neville-Jan 2003). This relates to what Dotson has called ‘testimonial smothering’ (Dotson 2011; Sakakibara 2023). The concern for such patients is not that an isolated piece of information will be dismissed as irrelevant, but that a broader judgement will be made regarding their competence as an interlocutor which could influence further investigations or decisions regarding their care.

Systematic gaps in medical knowledge, as well as inadequacies in medical frameworks (for example, Goldberg 2017) noted above, can give rise to ill persons’ perceived need to pre-emptively contort their reports of experience in order to present themselves in a way that they think will be palatable for their clinician(s). In practice, we see an interweaving of innocent and morally culpable errors. Inattentiveness and epistemic failure arising from the busy nature of healthcare work and highly routinised and standardised interactions interweave with prejudice and unfair credibility deficits. One of the goals of the epistemic injustice in healthcare research programme is to tease apart these errors and identify what types of steers—interpersonally and institutionally—might mitigate them and ameliorate the harms they cause.

### Theoretical expansion

NNH argue that expansion of the concept of epistemic injustice has introduced unwelcome fuzziness, suggesting that the expansion of epistemic injustice in healthcare research violates Fricker’s preferred “continued strictness” with the concept of testimonial injustice (p. 298). NNH are wary of new concepts such as epistemic microaggressions (p. 298), arguing that their breadth and reliance on the experience of the “victim” risks excessively broadening the concept of testimonial injustice.

NNH note that epistemic injustice in healthcare research deviates from Fricker’s original 2007 analysis in its focus, since Fricker’s original cases were focussed on socially marginalised groups along gender, race and class lines. This is significant because, they state, ill people exhibit diminished epistemic abilities which is not true for the groups Fricker was interested in. This, they argue, is insufficiently acknowledged in the literature (p. 298). They write: “unlike the groups described in Fricker’s work, who have no diminished epistemic abilities (i.e., abilities to provide testimony, pose questions, assert prior events, make value judgements, etc.), the situation can be different in healthcare.” (ibid.).

We agree that the possibility of diminished epistemic capabilities in vulnerable groups is important and warrants further attention. In the context of healthcare research, this becomes increasingly important as research is now routinely expected to involve ‘experts by experience’ or those with ‘lived experience’. However, it is important to consider why these perspectives are important and what the claims about them are. First, the claims about the significance of lived experience is not that each and every piece of information offered will be accurate or true. Such reports simply provide the ‘what it is like for me’ perspective about a particular situation or health state, and hence provide unique and significant information. These reports cannot be criticised for being subjective, because they are such by definition. For example, many people are anxious about going ‘into’ a CT scanner, despite the fact that the scanner is open and the test is not invasive. The epistemic contribution such accounts make is not to reveal that lay people misunderstand what a scanner is, but that they experience the test as uncomfortable and stressful. This is important from a service provision perspective. Such information cannot be gleaned in any other way. If we want to know how people feel about a particular intervention, medical procedure, or healthcare encounter, the only way to find out is by asking them. In this sense, such accounts are authoritative and have a distinctive epistemic contribution to make.

Second, we do not claim that ill people do not have epistemic limitations *tout court* but that local or temporary limitations (e.g. the effects of a sedative or slow recovery from head injury) can be perceived as broader limitations that are permanent, pervasive, or systemic. Careful attention needs to be paid to what limits are putative and what are to be expected within the context of a particular illness or medication. This is also true for the type of limitation, its duration and nature: is it transient or permanent? Does it affect overall cognition or just short-term memory? Such nuance is essential if our goal is to support and preserve epistemic agency as much as is possible (Carel and Kidd 2021; Carel and Gyoffry 2014). In paediatrics, for example, it is essential to understand the epistemic limits of developmental stages in order to resist a general and unjust dismissal of children’s perspectives, due to them being ‘irrational’ or ‘unable to tell fact from fiction’ (Carel and Gyoffry 2014).

This does not mean that every patient’s claim to epistemic injustice is correct. It does, though, invite us to identify what it is about a situation or encounter that left the person feeling dismissed, belittled or unheard. A high volume of such complaints, predating the concept of epistemic injustice by decades, is an indication of a dissatisfaction about the care and the kind of human encounters typical within healthcare that ought to be identified, documented, catalogued, and addressed (Carel 2018).

Third, we do not think that the contrast between ill persons and gendered/racialised/ class-marked individuals is as clear as NNH suggest. Indeed, work on hermeneutic and structural injustice (which NNH do not acknowledge in their paper) has been exploring these complexities for nearly two decades. For instance, it has been argued that structural injustices against people of low socio-economic status can give rise to epistemic injustice (Anderson 2012): this is not simply a matter of being disadvantaged by the prejudices held by others, but about being disadvantaged by one’s lack of access to certain markers of credibility (even if those markers are not inherently problematic, such as being literate). In other words, one’s epistemic contribution can already be compromised before the role of individually-held prejudice come into play (see Sect.  3.2).

While by no means an exhaustive treatment of this topic, there is a robust body of work that addresses the epistemic limitations of ill people in both somatic medicine and psychiatry (Byrne 2020, 2022; de Boer 2021; Harcourt 2021; Byrne and de Boer 2025; Dings and Strijbos 2025). Spencer et al. (2023). Spencer and Carel (2021) explore how the double bind of trivialisation and stigmatisation works to obscure the nature of some disorders, reducing sufferers’ capacity to describe their condition and gain credibility and support. Kidd and Carel (2018a, 2019, 2021) develop the notion of ‘the predicament of patients’ to articulate the epistemic harms and difficulties encountered by those who are somatically ill. How to respect the epistemic authority of those who are in the grip of psychosis, or experiencing paranoid delusion, or those who have been traumatised, has been the subject of philosophical attention (Houlders et al. 2021; Bortolotti et al. 2024; Bortolotti 2025). In addition, specific work on epistemic injustice in paediatrics, psychiatry and gerontology has attempted to distinguish between epistemic limitations that are the result of illness (e.g. being unconscious as the most extreme example) and epistemic limitations presumed by hearers due to stigma (‘all mentally ill people suffer from reduced capacity for reality testing’) (e.g. Chapman and Carel 2021). Further work aimed at distinguishing symptoms from developmental stage in children (Carel and Gyorffy 2014) and similar work on older people and on those with learning disabilities has followed (Chapman and Carel 2021).

## Conclusion

This paper has responded to a number of challenges raised by NNH in their paper. We have shown that applying standards of biomedical clinical trials on epistemic injustice in healthcare research is a mistake that unnecessarily applies criteria and methods that are inappropriate to the subject matter. We contend that ongoing empirical work into epistemic injustice in healthcare is both needed and being carried out, and support the call for more such research. Yet, there is an important difference between bolstering theoretical claims with empirical work and imposing rigid methodologies imported from the biomedical sciences onto complex social phenomena. Asserting that the phenomena’s resistance to being captured by such methodology undermines the phenomena’s existence is a mistake. Rather, phenomena like epistemic injustices require a methodologically pluralist approach to research.

## Footnotes

1 The EPIC Research Project| Epistemic Injustice in Healthcare [accessed on 24.4.2026].

2 Guro Engvig Løseth, presentation to the Nordic Winter Brain conference on 24.03.2026 entitled “Epistemic injustice and existential isolation in chronic pain and fatigue”.

3 See Martha’s Rule on the NHS website https://www.england.nhs.uk/patient-safety/marthas-rule/ [Accessed 21 March 2026].

4 Against “Epistemic Injustice” - by Robin McKenna [accessed on 23.4.26].

5 See the details of the case studies undertaken on the Project EPIC website https://epistemicinjusticeinhealthcare.org/

6 See the case studies on paranoia (https://epistemicinjusticeinhealthcare.org/prejudicing-paranoia) and dementia (https://epistemicinjusticeinhealthcare.org/discounting-dementia). 
